# Omicron infections profile and vaccination status among 1881 liver transplant recipients: a multi-centre retrospective cohort

**DOI:** 10.1080/22221751.2022.2136535

**Published:** 2022-11-04

**Authors:** Ensi Ma, Jingwen Ai, Yi Zhang, Jianming Zheng, Xiaogang Gao, Junming Xu, Hao Yin, Zhiren Fu, Hao Xing, Li Li, Liying Sun, Heyu Huang, Quanbao Zhang, Linlin Xu, Yanting Jin, Rui Chen, Guoyue Lv, Zhijun Zhu, Wenhong Zhang, Zhengxin Wang

**Affiliations:** a Department of General Surgery, Huashan Hospital, Fudan University, Shanghai, People’s Republic of China; b Department of Infectious Diseases, Huashan Hospital, Fudan University, Shanghai, People’s Republic of China; c National Medical Center for Infectious Diseases, Shanghai, People’s Republic of China; d Department of Organ Transplantation, Shanghai Changhai Hospital, Second Military Medical University, Shanghai, People’s Republic of China; e Department of General Surgery, Shanghai General Hospital, Shanghai Jiao Tong University School of Medicine, Shanghai, People’s Republic of China; f Organ Transplant Center, Shanghai Changzheng Hospital, Second Military Medical University, Shanghai, People’s Republic of China; g Liver Transplantation Center, Department of General Surgery, Shanghai Ruijin Hospital, Shanghai Jiao Tong University School of Medicine, Shanghai, People’s Republic of China; h Liver Transplantation Center, National Clinical Research Center for Digestive Diseases, Beijing Friendship Hospital, Capital Medical University, Beijing, People’s Republic of China; i Clinical Center for Pediatric Liver Transplantation, Capital Medical University, Beijing, People’s Republic of China; j Department of Hepatobiliary and Pancreatic Surgery, The First Hospital of Jilin University, Changchun, People’s Republic of China

**Keywords:** Omicron, SARS-CoV-2, liver transplantation, inactivated vaccine, protection rate, adverse events

## Abstract

A wave of Omicron infections rapidly emerged in China in 2022, but large-scale data concerning the safety profile of vaccines and Coronavirus disease 2019 (COVID-19) infection features in liver transplant (LT) recipients have not been collected. Therefore, the aim of this study was to assess the protectiveness and safety profile of the inactivated vaccines in LT patients against severe acute respiratory syndrome coronavirus 2 (SARS-CoV-2) Omicron variant infections. A multi-centre retrospective study was conducted in a cohort with a history of liver transplantation. A total of 1881 participants (487 vaccinated and 1394 unvaccinated patients) were enrolled from seven centres in China. Fourteen of the participants were infected by Omicron, and 50% patients had over 14 days of viral shedding duration. The protection rate of COVID-19 vaccinations to Omicron was 2.59%. The three breakthrough infections occurred more than 6 months after fully vaccinated. A total of 96 (19.7%) vaccinated patients had adverse events, including fatigue, myalgia, liver dysfunction, swelling, and scleroma. There were more Grade 3 adverse events in the preoperative vaccination group than those in the postoperative vaccination group. Inactivated whole-virion SARS-CoV-2 vaccines are safe in patients with post-liver transplantation. The efficacy of inactivated vaccines decreases after 6 months of vaccination, it is recommended that liver transplant patients get boosted vaccinations as early as possible even when they are fully vaccinated. Although clinical manifestations of Omicron infections were mild in LT patients, unvaccinated patients might have a higher risk of liver dysfunction during infections.

## Introduction

Coronavirus Disease 2019 (COVID-19), caused by the severe acute respiratory syndrome coronavirus 2 (SARS-CoV-2), has resulted in 0.53 billion infections and 6.3 million fatalities worldwide by 16th June 2022 [1]. A wave of Omicron infections rapidly emerged in China in 2022. The outbreaks were centred in Shanghai, Jilin and Beijing, while sporadic cases were detected in several other cities. The genomes of the viruses that caused the Omicron wave in China clustered with the SARS-CoV-2 BA.2 sub-lineage [2, 3]. Evidence suggests that patients with comorbidities are more susceptible to Omicron infection and progression to severe infections, especially solid organ transplantation recipients [4–7].

COVID-19 vaccination is one of the most effective ways to prevent COVID-19 infections and reduce decrease severity and mortality rate [8–11]. Evidence shows that fully/booster vaccinated individuals were substantially protected against COVID-19 [12, 13]. However, patients with decompensated cirrhosis in eastern China largely remained at unvaccinated, particularly those with hepatic encephalopathy and liver transplant (LT) [14]. In addition, recent studies have shown that COVID-19 vaccinations result in an impaired or poor humoral response in people with chronic liver diseases [15–18]. Therefore, vaccination promotions and investigations of COVID-19 infection prognosis in LT patients are urgently needed. However, large-scale data concerning the safety profile of COVID-19 vaccines in LT patients in China have not yet been collected. Additionally, the features of Omicron infections among these high-risk populations need to be elucidated. Thus, we conducted a multi-centre retrospective cohort study in China to illustrate the Omicron infection profile and vaccination status in LT patients.

## Methods

### Study design and patient enrollment

In our multi-centre cohort study, participants with a history of LT were enrolled from seven centres located in Shanghai, Beijing, and Jilin province, including Shanghai Huashan Hospital, Beijing Friendship Hospital, The First hospital of Jilin University, Shanghai Changhai Hospital, Shanghai First people’s hospital, Shanghai Ruijin Hospital and Shanghai Changzheng Hospital. All transplanted livers were matched by the China Organ Transplant Response System (COTRS) and obtained through Organ Procurement Organizations (OPO) from cardiac/brain death donors or living donor. The inclusion criteria are participants who had undergone LT previously, and the exclusion criteria is participants who were inability to provide informed consent. The study protocol was designed and informed consent obtained in accordance with the Helsinki Declaration and approved by the ethical committee of Huashan Hospital (KY2022-693).

Electronic questionnaires were used to collect data on demographics, history of LT, vaccination status, and COVID-19 infection characteristics until June 16, 2022. Data were collected on the etiology of liver disease, transplant date, immunosuppressant usage, drug concentration, and related laboratory testing. Comorbidities were also collected including hypertension, diabetes, hyperlipidemia, hyperuricemia, cardiovascular disease, cerebrovascular disease, respiratory disease, rheumatic immune disease and renal diseases, hypoleukocytemia and hematological diseases.

The SARS-CoV-2 infection was confirmed by SARS-CoV-2 real-time reverse transcription-polymerase chain reaction (RT–PCR) tests. The clinical course classification was evaluated according to the ninth National COVID-19 guidelines. Viral shedding duration was defined as the number of days from the date of the first positive PCR test result to the date of the first negative PCR test result followed by consecutive negative results. Additionally, we obtained epidemiological information of LT patients and analyzed their chest computed tomography (CT), liver function, respiratory support, and hospitalization days.

### Vaccination status and safety assessment

The vaccine information included number of doses, vaccine type, injection time, and adverse events post vaccination. Vaccine protection rate was calculated based on incidence density, and the 95%CI was calculated by calculator(http://www.openepi.com/PersonTime2/PersonTime2.htm). The questionnaire collected where the patients lived during the outbreak of Omicron, so we used the time when Omicron was first reported in each province as the starting point for observation (The exact outbreak time of each province is determined based on a combination of the outbreak notification from the Health Care Commission and the sequencing information released by each province – http://www.nhc.gov.cn/xcs/yqtb/list_gzbd.shtml). In addition, as the titres of antibodies produced by partially vaccination are too weak to produce effective immunity; and it takes some time (usually 14 days) from the fully vaccination to produce effective antibody titres. So, we divided the patients into two groups, the non/partially vaccinated group, and the fully/boosted vaccinated group. And 14 days after the fully vaccinated was used as the time when observation starts (Note: if the time was earlier than the outbreak time in the local province, the outbreak time was used as the start of observation). The adjusted HR were also calculated by Cox regression analysis. The safety assessment included profiling local (swelling and scleroma) and systemic adverse events (AEs) (fever, cough, fatigue, myalgia, nausea, and diarrhea). According to our previous reports [16, 17], abnormal liver function was defined as having at least one of the following liver function parameters: total bilirubin (>17.1 mol/L), alanine aminotransferase (ALT, >40 U/L), aspartate aminotransferase (>40 U/L), and *γ*-glutamyl transpeptidase (GGT, >50 U/L).

### Statistical analyses

If the continuous variables had a Gaussian distribution, the Kolmogorov–Smirnov test was used to assess significant differences in mean and standardized deviation values. Otherwise, the median and interquartile ranges were calculated. Categorical variables were presented as counts and percentages. Independent binomial variables were evaluated using Pearson’s Chi-Square test. Comparisons between two groups were performed using a Student’s *t*-test or Mann–Whitney *U* test, as appropriate. Statistical significance was set at *p* < 0.05. Statistical analysis was performed using the SPSS (version 20.0) software. Figures were generated using GraphPad Prism (version 8).

## Results

### Baseline characteristics

A total of 1881 patients who underwent LT were enrolled in this study. The overall infection rate was 0.7% (14/1881); however, higher rates of infections were found in cities with outbreaks. For example, the infection rates were 3.2% (7/218) in patients living in Shanghai and 2.4% (4/169) in patients living in Changchun, Jilin Province. The patients were divided into two groups according to their vaccination status. The vaccinated group had a median age was 45.2 years with 371 (76.2%) male patients. The number of patients partially, fully, and booster vaccinated were 38 (7.8%), 286 (58.7%) and 163 (33.5%) respectively. A total of 479 (98.4%) patients were vaccinated with inactivated vaccine (CoronaVac/BBIBP-CorV), 9 (1.8%) patients were vaccinated with recombinant protein vaccine (ZF2001) (1 as a booster vaccine after 2 doses of inactivated vaccine). 1 (0.2%) patient was vaccinated with adenovirus vaccine (CanSino) as a booster vaccine following 2 doses of inactivated vaccines. The baseline characteristics are summarized in Table 1.

**Table 1.** Baseline characteristics LT patients by vaccination status.

	Unvaccinated group (*n* = 1394)	Vaccinated group (*n* = 487)	*P* value
*Number of patients, n (%)*			
Shanghai Huashan Hospital	760 (75.8)	243 (24.2)	*** ***
Beijing Friendship Hospital	276 (71.7)	109 (28.3)	*** ***
The first hospital of Jilin University	212 (71.4)	85 (28.6)	*** ***
Shanghai Changhai Hospital	80 (79.2)	21 (20.8)	*** ***
Shanghai first people’s hospital	31 (79.5)	8 (20.5)	*** ***
Shanghai Ruijin Hospital	16 (59.3)	11 (40.7)	*** ***
Shanghai Changzheng Hospital	19 (65.5)	10 (34.5)	*** ***
*Number of patients, n (%)*			
Shanghai	171 (78.4)	47 (21.6)	*** ***
Beijing	60 (66.7)	30 (33.3)	*** ***
Changchun	115 (68.0)	54 (32.0)	*** ***
Others	1048 (74.6)	356 (25.4)	*** ***
*Age (years), mean ± SD*	42.1 ± 21.6	45.2 ± 19.3	***0***.***003***
*Gender, Male (%)*	990 (71.0)	371 (76.2)	***0***.***028***
*Comorbidity, n (%)*			
Diabetes	237 (17.0)	74 (15.2)	0.356
Hypertension	202 (14.5)	70 (14.4)	0.950
Hyperlipidemia	88 (6.3)	41 (8.4)	0.113
Hyperuricemia	143 (10.3)	54 (11.1)	0.607
Cerebrovascular disease	28 (2.0)	4 (0.8)	0.123
Cardiovascular disease	28 (2.0)	16 (3.3)	0.109
Respiratory diseases	22 (1.6)	11 (2.3)	0.325
Rheumatic immune disease	6 (0.4)	2 (0.4)	1.000
Renal diseases	37 (2.7)	10 (2.1)	0.465
Hematological diseases	10 (0.7)	1 (0.2)	0.352
Hypoleukocytemia	56 (4.0)	12 (2.5)	0.114
*Malignant tumour, n (%)*	318 (22.8)	126 (25.9)	0.171
*Prognosis of malignant tumour, n (%)*			
Recurrence and metastasis-free	235 (73.9)	107 (84.8)	***0***.***013***
Intrahepatic recurrence	49 (15.4)	10 (7.9)	
Pulmonary metastasis	22 (6.9)	4 (3.2)	
Osseous metastasis	11 (3.5)	0 (0)	
Other	16 (5.0)	9 (7.1)	
*Targeted/chemotherapeutic drugs, n (%)*	132 (56.1)	38 (30.2)	***0.027***
*Vaccination status, n (%)*			
Partially vaccinated		38 (7.8)	
Fully vaccinated		286 (58.7)	
Booster vaccinated		163 (33.5)	
*Vaccine type, n (%)*			
Inactivated vaccine		479 (98.4)	
Recombinant protein vaccine		9 (1.8) (1 as Booster)	
Adenovirus vaccine		1 (0.2) (1 as Booster)	
*Duration of initial inoculation to LT*			
Preoperative, *n* (%)		119 (24.4)	
Postoperative, *n* (%)		368 (75.6)	
*Infection rate (%)*			
Overall	0.8	0.6	0.939
With Malignant tumour	1.3	0.8	0.676
Without Malignant tumour	0.7	0.6	0.841
With Targeted/chemotherapeutic drugs	0.2	0.3	0.534
Without Targeted/chemotherapeutic drugs	0.1	0	0.829
Preoperative vaccination		1.7	
Postoperative vaccination		0.3	

*n*: number; SD: standard deviation.

### Vaccination status of LT patients by age groups

**Figure 1.**
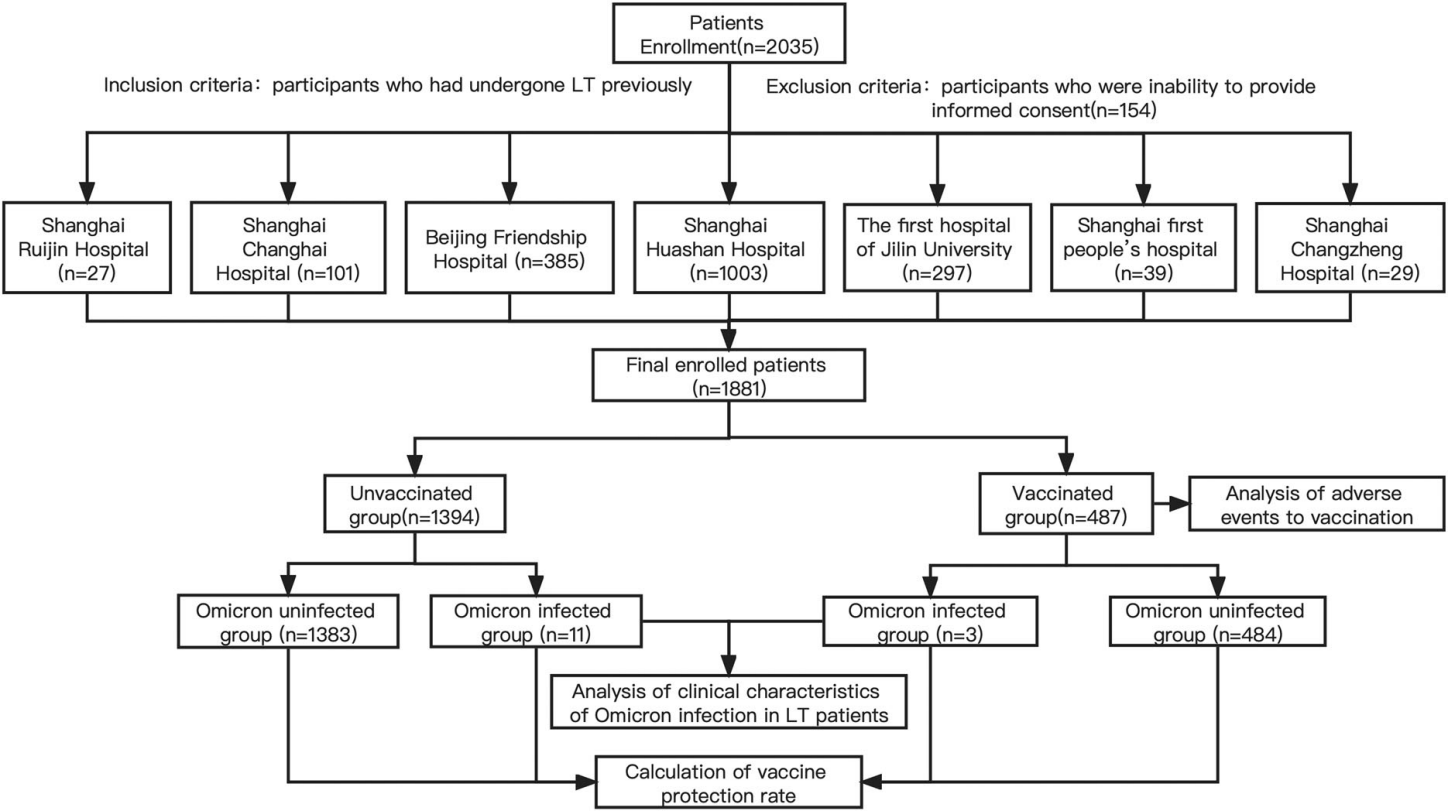
Flow chart of this study.

Unvaccinated patients comprised the majority of all age groups, but vaccination status varied among different age groups. Patients aged 18–39 years had the highest overall vaccination rate, and more than half of them were partially vaccinated. In addition, we found that the proportions of patients administered with booster vaccinations increased with age. Specifically, 0.50% (2/404) patients aged 18 years and younger received booster vaccinations, whereas the proportions increased progressively in the 18–39 age group, finally reaching the highest proportion of 8.48% (15/177). The proportions of vaccinated patients aged 40–60 years and those older than 60 years were 10.86% (102/939) and 12.19% (44/361), respectively. Details are shown in Figure 1.

### Adverse events of vaccination in LT patients

Adverse events occurred in 96 (19.7%) patients. The vaccinated patients were divided into groups based on the sequence of vaccination and liver transplantation (namely, preoperative and postoperative vaccination groups), presence of malignant tumours, and use of targeted or chemotherapy drugs. The adverse events outcomes of COVID-19 vaccination in patients who underwent LT are shown in Table 2. The most common adverse events in all groups were fatigue, myalgia, liver dysfunction, swelling and scleroma. The number of adverse events in the preoperative and postoperative vaccination groups were 37 (31.1%) and 59 (16.0%), respectively.

**Table 2.** Adverse events after vaccination.

Characteristics	Overall	Preoperative vaccination	Postoperative vaccination	Without malignant tumour	With malignant tumour	With targeted/chemotherapeutic drugs	Without targeted/chemotherapeutic drugs
	Patients (*n* = 487)	Patients (*n* = 119)	Patients (*n* = 368)	Patients (*n* = 361)	Patients (*n* = 126)	Patients (*n* = 38)	Patients (*n* = 88)
Total adverse events, *n* (%)	96 (19.7)	37 (31.1)	59 (16.0)^a^	74 (20.5)	22 (17.5)	8 (21.1)	14 (15.9)
Grade 3, *n* (%)	12 (2.5)	12 (10.1)	0 (0.0)^a^	7 (1.9)	5 (4.0)	2 (5.3)	3 (3.4)
*Local adverse events*							
Swelling and scleroma, *n* (%)	16 (3.3)	5 (4.2)	11 (3.0)	13 (3.6)	3 (2.4)	0 (0.0)	3 (3.4)
*Systemic adverse events*							
Fatigue, *n* (%)	55 (11.3)	25 (21.0)	30 (8.2)^a^	42 (11.6)	13 (10.3)	6 (15.8)	7 (8.0)
Myalgia, *n* (%)	57 (11.7)	24 (20.2)	33 (9.0)^a^	44 (12.2)	13 (10.3)	6 (15.8)	7 (8.0)
Nausea, *n* (%)	8 (1.6)	2 (1.7)	6 (1.6)	8 (2.2)	0 (0.0)	0 (0.0)	0 (0.0)
Fever, *n* (%)	8 (1.6)	4 (3.4)	4 (1.1)	7 (1.9)	1 (0.8)	0 (0.0)	1 (1.1)
Diarrhea, *n* (%)	6 (1.2)	3 (2.5)	3 (0.8)	5 (1.4)	1 (0.8)	1 (2.6)	0 (0.0)
Cough, *n* (%)	4 (0.8)	2 (1.7)	2 (0.5)	4 (1.1)	0 (0.0)	0 (0.0)	0 (0.0)
Epistaxis, *n* (%)	1 (0.2)	0 (0.0)	1 (0.3)	1 (0.3)	0 (0.0)	0 (0.0)	0 (0.0)
Ileus, *n* (%)	1 (0.2)	0 (0.0)	1 (0.3)	1 (0.3)	0 (0.0)	0 (0.0)	0 (0.0)
Ear chills, *n* (%)	1 (0.2)	1 (0.8)	0 (0.0)	0 (0.0)	1 (0.8)	0 (0.0)	1 (1.1)
Scalp blister, *n* (%)	1 (0.2)	0 (0.0)	1 (0.3)	1 (0.3)	0 (0.0)	0 (0.0)	0 (0.0)
Liver dysfunction, *n* (%)	27 (5.5)	15 (12.6)	12 (3.3)^a^	19 (5.3)	8 (6.3)	3 (7.9)	5 (5.7)

^a^ Means that the difference was statistically significant, compared with the corresponding.

It was a higher rate of grade 3 adverse events in the group of preoperative vaccination, than that in postoperative vaccination group. All 12 patients who developed grade 3 adverse events were in the preoperative vaccination group, and five patients were also with malignant tumours. In contrast, all adverse events were mild and self-limiting in the postoperative vaccination group. These results suggest that extra caution should be exercised in administering preoperative vaccinations for LT patients with malignant tumours, although a larger sample size is still needed to verify this conclusion.

### Vaccine effectiveness in LT patients

The infection rate was 0.79% (11/1394) in the unvaccinated group and 0.62% (3/487) in the vaccinated group. Owing to the sample size and short follow-up time, we calculated the vaccine protection rate based on incidence density between non/partially vaccinated group and the fully/boosted vaccinated group, which was 2.59% (95% CI: −249.10% to 72.82%). Moreover, Cox regression analysis resulted in an HR value of 0.962 (95% CI: 0.267–3.466, *p* = 0.953) for the fully/boosted vaccinated group compared with the non/partially vaccinated group. Notably, the three patients who had breakthrough infections were all fully vaccinated, but their fully vaccination time was 2021/9/9, 2021/8/31 and 2021/8/15, while the infection time was 2022/3/28, 2022/4/9 and 2022/4/3 respectively. It can be seen that the infections occurred more than 6 months after fully vaccinated, and this correlates with the fact that the efficacy of inactivated vaccine decreases substantially after 6 months of vaccination.

### Characteristics of Omicron infected LT patients

14 patients in this study were diagnosed with Omicron infection. All the infected LT patients included in this study had mild clinical manifestations, which were diagnosed according to the latest ninth edition of the national COVID-19 guidelines. None of the patients reported respiratory distress during the treatment nor received oxygen treatment. Only 1 patient was administered paxlovid. Notably, 3 patients had liver dysfunction during treatments, which were all in the unvaccinated group (3/11, 27.3%). None of the vaccinated patients developed liver dysfunction after breakthrough infections. Although the sample size is small, the limited data shows that vaccination may prevent the worsening of symptoms after Omicron infection in LT patients. The relevant content is summarized in Table 3.

**Table 3.** Clinical characteristics of Omicron infected and uninfected patients.

Characteristics		Number of infected patients(*n* = 14)	Number of uninfected patients(*n* = 1867)
*Baseline characteristics*			
Age, *n* (%)			
	<18y	2 (14.3)	402 (21.5)
	18–39y	7 (50.0)	153 (8.2)
	40–60y	3 (21.4)	953 (51.0)
	>60y	2 (14.3)	359 (19.2)
Gender, *n* (%)			
	Male	9 (64.3)	1352 (72.4)
	Female	5 (35.7)	515 (27.6)
Vaccination status, *n* (%)			
	No	11 (78.6)	1383 (74.1)
	Yes	3 (21.4)	484 (25.9)
Comorbidity, *n* (%)			
	No	9 (64.3)	1053 (56.4)
	Yes	5 (35.7)	814 (43.6)
Malignant tumour, *n*(%)			
	No	9 (64.3)	1428 (76.5)
	Yes	5 (35.7)	439 (23.5)
Targeted/chemotherapeutic drugs, *n* (%)			
	No	2 (40.0)	272 (62.0)
	Yes	3 (60.0)	167 (38.0)
Characteristics during hospitalization			
Clinical type, *n* (%)			
	Mild	14 (100.0)	/
Oxygen inhalation during hospitalization, *n* (%)			
	No	14 (100.0)	/
Liver dysfunction during hospitalization, *n* (%)			
	No	11 (78.6)	/
*** ***	Yes	3 (21.4)	/
Paxlovid during hospitalization, *n* (%)			
	No	13 (92.9)	/
	Yes	1 (7.1)	/
Days of viral shedding, *n* (%)			
	≤7d	3 (21.4)	/
	8-14d	4 (28.6)	/
	>14d	7 (50.0)	/

*n*: number.

### Factors influencing viral shedding in LT patients

**Figure 2.**
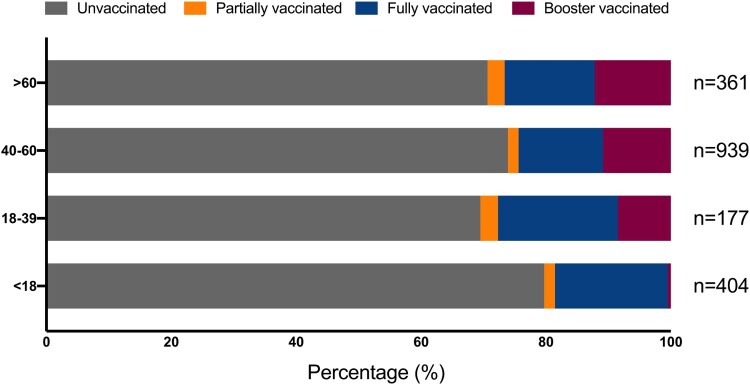
Proportion of LT patients vaccinated by age group.

Data from Japan showed that Omicron could not be detected in samples after 10 days since diagnosis or symptom onset [19]. In China, the median duration of the viral shedding by Omicron is 11.13 days in non-severe patients [20]. However, the present study showed that LT patients had a longer duration of viral shedding, with a median duration of 14 days. The details are shown in Figure 2(B).

**Figure 3.**
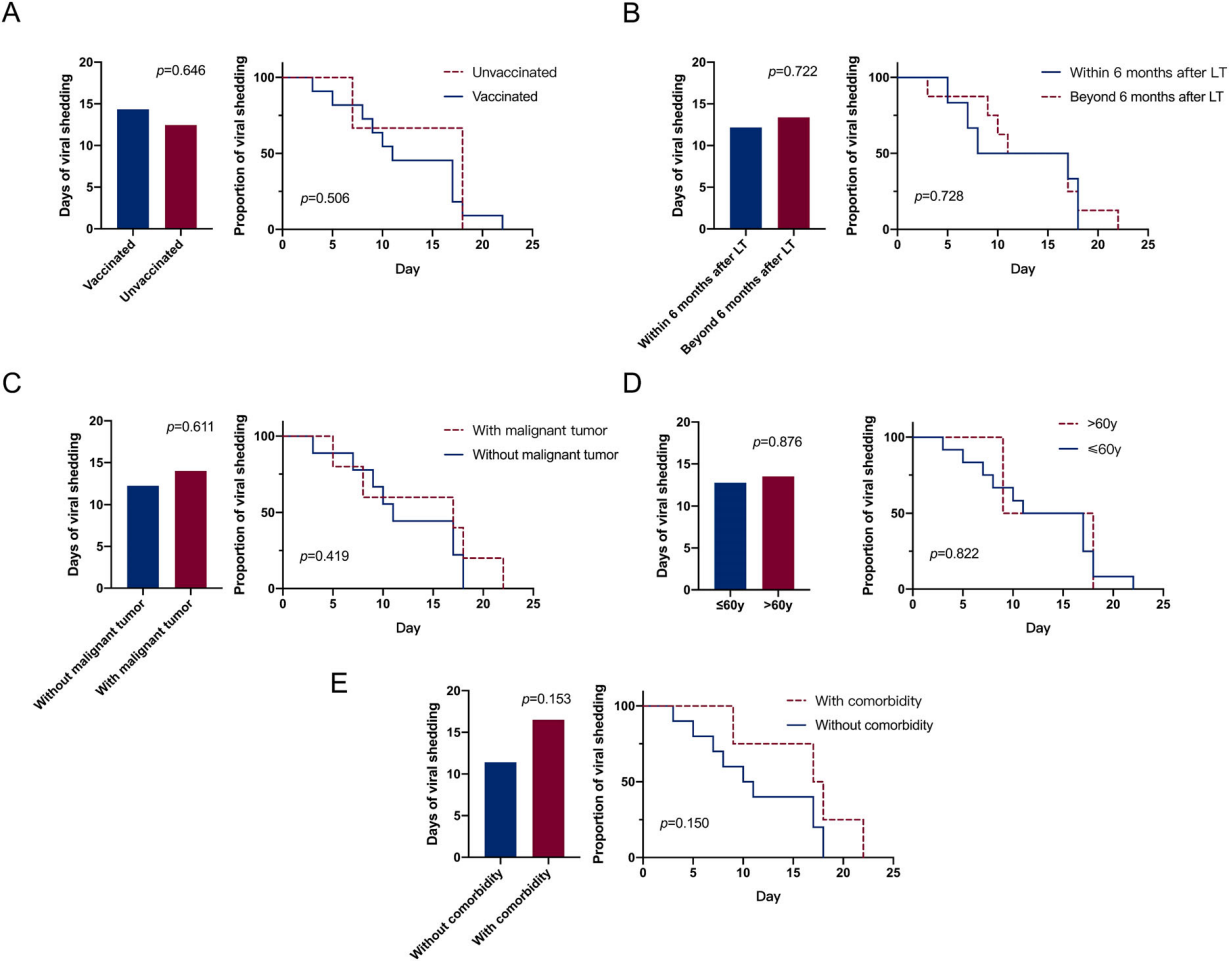
Effects of vaccination status (A), time after LT (B), malignant tumour status (C), age (D) and comorbidity (E) on the time of viral shedding.

A subgroup analysis was performed on 14 patients with Omicron infections. The analysis revealed that once the LT patients were infected, the duration of viral shedding was not significantly correlated with the inoculation of vaccination, presence or absence of malignant tumours, age, post-surgery duration, and comorbidities (Figure 3(A–E)).

## Discussion

The local infection rate was 2.4% [2] in Shanghai and 0.6% [21] in Changchun, which were largely different from the infection rate observed in the LT patient cohort of the present study. This can be partly attributed to the fact that LT patients are more susceptible to infection than the healthy population due to immunosuppression, indicating a more important role of COVID-19 vaccinations in LT patients. And we found patients aged 18–39 years had the highest overall vaccination rate, this can be partly attributed to the fact that relatively high participation and motivation in society of patients in this age group and they do not have some conditions that would make them inappropriate for vaccination, unlike children and the elderly.

Several studies have evaluated the efficacy and responsiveness of the vaccinations in LT patients and demonstrated low response rates ranging from 47% to 80% [22–25]. However, the mortality rate of breakthrough infections in vaccinated LT patients is lower than that in unvaccinated patients [26]. Undeniably, a series of adverse events may occur after vaccination, with LT patients experiencing mild adverse events, more severe liver damage [27], and moderate or severe liver allograft rejection after vaccination [28, 29].

Recently, Saharia K. K. [30] suggested that booster vaccinated is effective against SARS-CoV-2 variants, except for Omicron. However, all of the above studies focused on mRNA vaccines. Prieto J. [31] investigated the protective effect of inactivated vaccines in 11 LT patients, Tu Z. H. [15] evaluated the safety of inactivated vaccines in 35 LT patients. Data on the safety and protection of inactivated vaccines in large-scale LT patients are not available, as yet. Although the data on inactivated vaccines in the LT population are not yet sufficient, studies show a decreased immunogenicity in patients with other chronic liver diseases (e.g. cirrhosis and fatty liver) compared to healthy individuals [16, 32–34].

Overall, inactivated COVID-19 vaccines were safe and well tolerated by LT patients in our study. Adverse events occurred in 96 (19.7%) patients. Local and systemic AEs were mostly mild, with fatigue being the most frequently detected systemic AE. Previous studies reported that 27%−51% vaccinated LT patients had systemic AEs (mostly fatigue and headache) following two doses of vaccines [15, 35, 36]. Compared with liver cirrhosis and other chronic liver diseases [16, 17], LT patients had a relatively higher proportion of systemic AEs, which might be due to their impaired immune systems.

Our study illustrated that the 14 infected LT patients had mild Omicron infections, and only 3 of them had received fully vaccination before being infected. Several studies have confirmed the severity and mortality of Omicron infections increased in immunosuppressed populations, including patients with malignancy [37], and lung transplant patients [38]. This suggests that LT patients require adequate protection against Omicron infections.

COVID-19 vaccinations are one of the most effective ways of preventing COVID-19 infections and reduce the severity and mortality rate [10, 11, 39]. A previous study found a 20% vaccine effectiveness (95% CI, −25%−49%) against noncritical Covid-19 infections in adolescents aged 12–18 years during the Omicron-predominant period [40]. Although LT patients are usually at a higher risk of immunosuppression, our study found that the protective rate of COVID-19 vaccinations against Omicron infections in LT patients was 2.59%. It can be seen that the protection rate of inactivated vaccine is relatively weak, and we think this is due to mainly two reasons: firstly, the organism immune status of liver transplant patients is inferior to that of healthy individuals, meanwhile there is a gap in the efficacy of inactivated vaccine compared to mRNA vaccine [41]. Notably, three fully vaccinated patients (3/286) had breakthrough infections, and the infections all occurred more than 6 months after fully vaccinated, this correlates with the fact that the efficacy of inactivated vaccine decreases substantially after 6 months of vaccination, meanwhile, no booster vaccinated patient (0/163) had breakthrough infections, though the data is limited, it still indicated that the booster vaccination was effective in improving immunity to Omicron, so it is recommended that liver transplant patients receive booster vaccines as early as possible to boost immunity even when they are fully vaccinated.

The infected LT patients that were previously vaccinated had mild symptoms and no liver dysfunction. As LT patients have a low immune function, they are expected to show a lower response to COVID-19 vaccines. One study showed that the antibody response of LT patients was partially or poor after one or two doses of vaccines; however, a third dose of vaccine could further improve the humoral immunity of these patients [42]. The limited effectiveness of the vaccines in LT patients may also be partially attributed to the fact that COVID-19 vaccines were not designed for Omicron variant. Therefore, there is an urgent need to develop vaccines against Omicron infections to improve the vaccine protection rate. Additionally, we found LT patients with comorbidities had prolonged viral shedding time compared with those without comorbidities. Patients with comorbidities were previously reported to have a higher severe infection rate previously [4]. Therefore, comorbidities of LT patients should also be considered in the future vaccinations.

There are several limitations with this study. First, patients included with a short follow-up period. Second, participants had different pre-transplant liver diseases; therefore, studies with larger sample size and more diverse pre-transplant liver diseases are required in the future. Third, breakthrough infections were observed in only a small number of patients; thus, a larger sample size is needed for risk analysis of the breakthrough infections. Moreover, T and B cell response tests or other immunological tests, and these tests were not performed in this study, which will be designed in future investigations. Last, the majority of the subjects received inactivated vaccines; therefore, the findings of this study may not be generalized to mRNA or other vaccines.

In conclusion, inactivated whole-virion SARS-CoV-2 vaccines are safe in patients with post-liver transplantation. The efficacy of inactivated vaccines decreases after 6 months of vaccination, it is recommended that liver transplant patients get boosted vaccinations as early as possible even when they are fully vaccinated. Although clinical symptoms of Omicron infections among LT patients are generally mild, regardless of their vaccination status; however, unvaccinated patients might have a higher incidence of liver dysfunction during infections.

## Supplementary Material

Supplemental Material
