# Supplementary material for: Omicron infections profile and vaccination status among 1881 liver transplant recipients: a multi-centre retrospective cohort
Source: Emerg Microbes Infect. 2022 Nov 4;11(1):2636–44. doi: 10.1080/22221751.2022.2136535 (PMC9639509; doi:10.1080/22221751.2022.2136535)
Supplement: Supplemental Material [file TEMI_A_2136535_SM5071.docx]

LT patients require immunosuppressants (the most common of which are tacrolimus and sirolimus), and some patients require antivirals because of viral hepatitis. Therefore, we classified the patients according to this immunosuppressive as well as antiviral drugs and calculated the vaccine protection rate according to the density, with the following results: 75.0% (1410/1881) of patients in this cohort were taking only tacrolimus, with a vaccine protection rate of 61.3%;14.5% (272/1881) of patients were taking only sirolimus, with a vaccine protection rate of - 506.3%; and 4.5% (85/1881) of patients were taking both tacrolimus and sirolimus, but the vaccine protection rate could not be calculated because no one was infected in this group of patients; 6.1% (114/1881) of patients were taking other immunosuppressive drugs, with a vaccine protection rate of 100%; 64.9% (1220/1881) of patients were taking antivirals, with a vaccine protection rate of -21.6%; 35.1% (661/1881) of patients not taking antivirals, with a vaccine protection rate of 43.8%. Based on the above data we can tentatively conclude that the vaccine had a higher protection rate in the subgroup of patients taking tacrolimus, while the use of sirolimus and antiviral drugs may reduce the protection rate of the vaccine. But due to the small sample size of these two subgroups, subsequent validation with a larger sample size is needed.

We further analyzed the cohort of 444 patients with combined malignancy (126 of whom were vaccinated). 119 of the 444 patients developed recurrence or metastasis, with only 9 after vaccination, 15 before vaccination and 95 without vaccination. Thus, the proportion of vaccinated patients who had recurrence or metastasis after vaccination was 7.1% (9/126), while the proportion without vaccination was 29.9% (95/318), which showed that vaccination did not promote tumor recurrence. In addition, for adverse reactions, among 487 vaccinated patients (126 of whom had liver malignancies), 12 had serious adverse reactions(Grade 3) after vaccination, including 5 with malignant tumors. The proportion of serious adverse reactions in patients with malignant tumors was 4.0% (5/126), while the proportion in patients without malignant tumors was 1.9% (7/361), p=0.21, which means the proportion of serious adverse reactions in patients with malignant tumors was not significantly higher than that in patients without malignant tumors.

Considering that LT patients require immunosuppressants (the most common of which are tacrolimus and sirolimus), in addition some patients require antivirals because of viral hepatitis. Therefore, we classified the patients according to this immunosuppressive as well as antiviral drugs and calculated the vaccine protection rate according to the incidence density, with the following results: 75.0% (1410/1881) of patients in this cohort were taking only tacrolimus, with a vaccine protection rate of 61.3%, 14.5% (272/1881) of patients were taking only sirolimus, with a vaccine protection rate of - 506.3%, and 4.5% (85/1881) of patients taking both tacrolimus and sirolimus, and the vaccine protection rate could not be calculated because no one was infected in this group of patients, 6.1% (114/1881) of patients were taking other immunosuppressive drugs, with a vaccine protection rate of 100%. 64.9% (1220/1881) of patients taking antivirals, with a vaccine protection rate of -21.6%, while 35.1% (661/1881) of patients not taking antivirals, with a vaccine protection rate of 43.8%. Based on the above data we can tentatively conclude that the vaccine had a higher protection rate in the subgroup of patients taking tacrolimus, while the use of sirolimus and antiviral drugs may reduce the protection rate of the vaccine, but due to the small sample size of these two subgroups, subsequent validation with a larger sample size is needed. For adverse events, we also made relevant comparisons and found 76.2% (371/487) of patients in this cohort were taking only tacrolimus, with a grade 3 adverse events rate of 1.9%, 14.6% (71/487) of patients were taking only sirolimus, with a grade 3 adverse events rate of 1.4%, and 3.5% (17/487) of patients taking both tacrolimus and sirolimus, with a grade 3 adverse events rate of 11.8%, 5.7% (28/487) of patients were taking other immunosuppressive drugs, a grade 3 adverse events rate of 7.1%(p=0.022). Since all 12 patients who had a serious adverse events were vaccinated before LT, and 487 of the patients included in this study were vaccinated, 119 of whom were vaccinated before surgery. According to age, gender, with/without malignant tumor, with/without comorbidity, with/without antiviral drugs, and with/without targeted/chemotherapeutic drugs, we performed propensity score matching (setting match tolerance to 0.03) in the 119 patients, and 11 patients were successfully matched. Among these 22 patients, we found that the age of patients in the group occurring serious adverse events was 52.8 ± 10.1 years, compared to 44.8 ± 16.3 years in the group not occurring (p=0.181). The proportion of males in the group occurring serious adverse events was 90.9% (10/11) and 90.9% (10/11) in the group not occurring (p=1.000). The proportion of patients with malignancy in the group occurring serious adverse events was 36.4% (4/11) compared to 18.2% (2/11) in the group not occurring(p=0.632). The proportion of patients with underlying disease in the group occurring serious adverse events was 63.6% (7/11) and 54.5% (6/11) in the group not occurring(p=0.665). The proportion of patients taking antivirals drugs was 90.9% (10/11) in the group occurring serious adverse events and 81.8% (9/11) in the group not occurring (p=1.000). The proportion of patients taking targeted/chemotherapy drugs was 9.1% (1/11) in the group occurring serious adverse events and 0% (0/11) in the group not occurring (p=1.000).
